# Young Children’s Inductive Inferences Within Animals Are Affected by Whether Animals Are Presented Anthropomorphically in Films

**DOI:** 10.3389/fpsyg.2021.634809

**Published:** 2021-06-03

**Authors:** Andrzej Tarłowski, Eliza Rybska

**Affiliations:** ^1^Institute of Psychology, University of Economics and Human Sciences in Warsaw, Warsaw, Poland; ^2^Laboratory of Nature Education and Conservation, Adam Mickiewicz University, Poznań, Poland

**Keywords:** anthropocentrism, anthropomorphism, inductive inference, experience, naïve biology

## Abstract

Children are exposed to anthropomorphized animals in a variety of contexts. The literature that analyzes this phenomenon suggests that exposure to anthropomorphic media may strengthen children’s anthropocentric representation of animals. There is an as yet unexplored difference between anthropomorphized and realistic depictions of multiple animal species presented simultaneously in films. The anthropomorphized animals all behave like humans, so they are more behaviorally similar to one another than animals depicted realistically. We asked whether witnessing multiple species depicted anthropomorphically or realistically influences the way 5-year-old children perceive internal commonalities among animals. One group of children (*n* = 37) watched a cartoon presenting multiple species of anthropomorphized animals, the other group (*n* = 38) watched a nature documentary that also presented multiple species. Both groups extended a novel internal feature from an animal to a variety of items including diverse animal species. Children watching a cartoon made significantly stronger projections to non-human animals than children watching the documentary. Children’s projections to humans and inanimate objects did not differ between the groups and were uniformly low. One of the possible explanations of the results is in terms of children’s essentialist expectation that behavior is caused by internal properties.

## Introduction

Acquiring accurate biological understanding is a challenging developmental process. It is a truism to say that experiences with the natural world facilitate the acquisition of biological knowledge. However, is it always the case that more experience translates into faster development of mature understanding? Experiences vary in effectiveness, with some even being detrimental. For example, students are often convinced that a snail may lose its shell, and direct observation of a slug without scientific knowledge about slugs and snails can enhance this misconception (e.g., Rybska and Sajkowska, 2012; Rybska, 2018). Primary school children often fail to delineate the boundaries of the living domain, most notably by excluding inanimate living things, such as plants and fungi (Anggoro et al., 2008). Opfer and Siegler (2004) show that children tend to include plants in the living domain once they learn that plants, like animals, can move toward goals, but they do not progress to the mature understanding, when informed that plants, like animals, grow and need water.

There is much interest in how direct and culturally mediated experiences differ in shaping biological knowledge (e.g., Duerden and Witt, 2010). While direct experience necessarily exposes children to living things as they really are, there is large variability in the degree of realism of culturally mediated representations. This realism in terms of children’s cultural content is a subject of study in its own right, with the discussion involving the effectiveness of fictitious, and unrealistic content as sources of knowledge about the world. The conclusion from this research is that unrealistic content is less conductive of learning than realistic content (Hopkins and Weisberg, 2017). The level of realism in culturally mediated representations, and its role in shaping biological thought, are the main focus of the present article, with the specific interest devoted to anthropomorphism as a way of distorting reality.

Anthropomorphism, that is, ascribing human qualities to non-human beings (Quinn et al., 2016), is a ubiquitous characteristic of current cultural representations of the natural world (Geerdts, 2016). Children’s books and films are replete with representations of animals that mimic humans (McCrindle and Odendaal, 1994). Even educational materials aimed at building biological knowledge often portray animals in ways that make them unrealistically similar to humans (Simcock and DeLoache, 2006, 2008; Simcock and Dooley, 2007). Due to its ubiquity, anthropomorphism’s place in children’s developing biological knowledge is a subject of considerable scrutiny. In her review, Geerdts (2016) points to both potentially positive and negative effects. For example, anthropomorphism may facilitate analogical transfer within the domain of living things. It may promote human to other animal analogical reasoning which, according to Inagaki and Hatano (2002), is an effective learning tool at early stages of development. The negative effects of anthropomorphism include wrongly attributing human features to other animals and strengthening children’s anthropocentric tendencies (Waxman et al., 2014).

Anthropocentrism is the expectation that humans are the most important organisms in the universe; and a tendency to interpret or regard the world in terms of human values and experiences, for example, an anthropocentrically minded child may expect that plants photosynthesize because they are hungry. Anthropocentrism also manifests itself in regarding humans as a privileged knowledge source in the living domain (Carey, 1985).

Early research (Carey, 1985) suggested that anthropocentrism is a developmental universal and the consequence of the way core knowledge is organized. Carey (1985) argued that core knowledge includes an early understanding of intentionality but it does not include any specifically biological understandings, such as an awareness of the role of internal organs in supporting metabolism, growth, and reproduction. By this account, knowledge about humans serves as a foundation for the understanding of the whole biological domain and therefore the possession of human-like features serves as a key criterion for the inclusion into the living domain. Anthropocentrism is manifested in reasoning in the form of asymmetries in inference that can be interpreted as prototypicality effects (Carey, 1985). When children extend novel features, inferences within animates are stronger from humans, a prototype living thing, than from atypical living things, namely, all non-human animals.

The characterization of anthropocentrism as a developmental universal has been challenged by research showing that the anthropocentric pattern of inferences is limited to a specific group of children. Childhood anthropocentrism has been linked to limited direct exposure to the natural world because it is observed solely in urban children (Ross et al., 2003; Medin et al., 2010). It has also been shown that even in urban populations, anthropocentrism emerges at around 5 years of age and is absent in younger children (Herrmann et al., 2010). Most importantly from the viewpoint of the present argument, the anthropocentric tendencies are strengthened by the exposure to anthropomorphic cultural representations of animals (Waxman et al., 2014).

Anthropocentrism is a key consequence of anthropomorphic depiction of animals (Waxman et al., 2014), but it is not the only one. There is evidence suggesting that the anthropomorphic portrayal of animals in educational materials leads to poor recall of specific biological facts (Geerdts et al., 2015), poor transfer of biological knowledge (Bonus, 2019), and unwarranted extension of human features to other animals (Ganea et al., 2014; Waxman et al., 2014). Ganea et al. (2014) manipulated anthropomorphism in verbal and pictorial descriptions of novel animals and tested, among other things, unwarranted projections of human features to animals. The results showed that 5-year-old children exposed to fully anthropomorphic material were more likely to make unduly anthropomorphic projections to animals compared to children exposed to fully realistic content.

In this article we want to examine other, so far unexplored, potential consequences of anthropomorphic portrayal of the biological world, namely, that depicting various animals as human-like increases the perception of their internal similarity and manifests itself in stronger inductive inferences. Many media depictions of animals present them all behaving like humans and having many human-like features. Anthropomorphic presentation of animals often degrades the information about real between-species differences, particularly those pertaining to behavioral dispositions. All the protagonists in anthropomorphic animal stories can talk, reason, play and dance as humans can. Between-species differences are often reduced to character-trait differences. Presenting diverse animal kinds as human-like may blur the real differences between species and create an impression that animals are more behaviorally and physiologically similar than they really are. This unifying depiction of animals may influence the development of biological thought and interfere with educational practices aimed at shaping the understanding of biology as a science. Previous research on the effect of anthropomorphic portrayal of animals did not include the unified portrayal of various species. For example, in Waxman et al.’s (2014) anthropomorphic condition, only one species, bears, was presented as human-like. In their research, children did not have the opportunity to make any comparisons between bears and other species of animals, or to adjust between-species similarity metrics. An interesting question can be asked: does the unifying portrayal of various animals as human-like affect the way children reason about real animals? We might hypothesize that one possible consequence of presenting varied species of animals as similar to humans is that children begin to weigh differences between species less in their reasoning. They may perceive diverse kinds of animals to be similar. This could make children less able to appreciate the significance of biodiversity and between-species differences, in particular. If this were the case, being subjected to unifying anthropomorphic cultural input about animals could lead to increased strength of inferences within the animate domain. If all animal species are seen as more like one another by children exposed to unifying anthropomorphic content, then those children would be more willing to attribute a novel biological feature learned on one animal to other kinds of animals.

Research comparing children with diverse experiences with the natural world suggests that rural children, who have more experience with biological diversity, tend to make projections that are more restricted (Tarlowski, 2006) and more based on biological similarity (Ross et al., 2003) than do urban children. Bartoszek and Rybska (2016), who asked urban and rural children to express their ideas about farm animals and pets, observed, among other things, that urban children pictured them in a similar, unifying way, with some elements of anthropomorphic perspective, where, for example, parrots were drawn with human-like mouth, the same way they were pictured on mammals. So far, however, no study has looked at the effect of being exposed to a varying degree of behavioral similarity on inductive inferences. Research with adults suggests that behavioral similarity does not inform inferences about internal features (Heit and Rubinstein, 1994). Adults use separate metrics of similarity for internal and behavioral feature projections. However, developmental studies show that infants and young children link insides and behavior (Newman et al., 2008; Setoh et al., 2013; Meyer et al., 2017). One way to reconcile this difference between adult and child studies has to do with theories that are available at different stages in development of biological knowledge, and how these theories affect perceptions of similarity in inductive inference. It could be argued that adults, and not children, are aware that some behavioral similarities, such as flight in bats and butterflies, stem from convergent evolution rather than kinship, thus rendering behavioral similarity less relevant to adults’ inferences about insides. In contrast, young children do not have a theoretical framework that would allow them to separate behavioral similarity from internal similarity. Instead, according to Gelman (2003), children’s understanding of animals is essentialist, which means that they expect all observable, kind specific features, including behavior, to originate from causally central, internal qualities.

In order to test whether the dimension of uniformity—diversity of animal behavior in anthropomorphic and realistic depictions affects the strength of projections within animates, we showed children two types of films, one presenting desert animals realistically, the other presenting desert animals anthropomorphically. We later asked children to extend an internal property of one animal to a diverse set of items including other animals. We expected that children who watched the anthropomorphic, behaviorally unifying portrayal of animals would generalize more broadly and indiscriminately than those who watched the realistic, behaviorally diverse portrayal of animals. We focused on 5-year-olds, because this is the age at which the effects of exposure to anthropomorphic content on inductive inferences has been documented (Waxman et al., 2014).

The unique feature of the present design is that it examines one of the most ubiquitous aspects of children’s cultural experience with the natural world, that is, the anthropomorphic films about animals, and it looks at how such films influence the formation of biological knowledge. Despite a large number of studies looking at the effect of experiences on induction, no research has looked at the influence of anthropomorphizing films.

In accordance with Kemp and Jern’s (2014) taxonomy of inductive problems, the method chosen to measure changes in children’ perception of animal categories is a single feature generalization. In this task, participants are presented with a novel feature of a single instance or a category and their task is to decide whether a variety of other instances or categories also has this feature.

## Methods

### Participants

The study group consisted of a convenience sample of 75 children who attended five private preschools in the Warsaw urban area. The children’s mean age was 69 months with the standard deviation of 4 months and a range between 60 and 81 months. There were 36 girls and 39 boys in the sample. Nineteen boys in the anthropocentric condition were 69 months old, *SD* = 4. Twenty boys in the realistic condition were 69 months old, SD = 5. 18 girls in the anthropomorphic condition were 68 months old, *SD* = 5, and 18 girls in nature documentary group were 67 months old, *SD* = 4. The preschools participating in the project had an expanded nature curriculum that included hands-on experience with living things and experimentation. We did not collect data on socioeconomic status from parents but it can be inferred from the location and type of preschool that it was high as Warsaw is a high SES municipality being among the highest median income communities in Poland. Moreover, children were recruited from private preschools which are tuition-based. Parents signed informed consent forms and they were presented with the description of the study. There was no payment for participation.

### Materials

Children were presented with two films depicting animals either anthropomorphically or realistically. One group of children watched an excerpt from an animated motion picture “Rango,” directed by Gore Verbinsky. The film presents a story of a chameleon who accidentally arrives at a desert town and, in the company of other animals, takes up the fight to regain stolen water. The second group of children watched a nature documentary, “Mysteries of desert dwellers: honey badger (ratel) and chameleon,” directed by Makoto Kita and Yoshiko Shinohara. Importantly, both films presented a variety of animal species. There were 24 animal species featured in the nature documentary excerpt with the most prominent roles played by chameleon, honey badger and giraffes. The animals included 12 mammals, five birds, four reptiles, and three arthropod species. The cartoon featured a larger number of species, but if the ones that appeared in the background are excluded, then the number is comparable to the nature documentary, with 25 species including humans, 11 other mammals, 5 birds, 3 reptiles, 2 amphibians, and 3 arthropod species. The chameleon was a dominant figure in the cartoon and he featured in almost all the scenes. Cross-species interactions were commonplace in the cartoon, while in the documentary, most species were presented in isolation, with rare exceptions, such as chameleon hunting a beetle, honey badger hunting a snake or interacting with a porcupine or a lion.

The base of inference in the induction task, the lizard, was presented as a photograph on an A4 sheet. This presentation was accompanied with the presentation of the target feature. The measure of inductive inference was obtained with the use of an A3 response sheet with a matrix of 30 photographs of items representing a broad variety of categories. The photographs included humans (woman and child), mammals (cow, sheep, mink), birds (duck, European robin, seal, heron), reptiles/amphibians (lizard, frog), invertebrates (grasshopper, butterfly, snail, sea cucumber), plants and fungi (tree, flower, mushroom, fern, moss), artifacts (lamp, phone, car, electric appliance, motorbike, stuffed animal), inanimate natural objects (fire, water, stones).

### Procedure

The study was carried out separately in each preschool. Children were randomly assigned to one of two groups. One of the groups watched an animated motion picture anthropomorphizing animals (anthropomorphic condition), the other group watched a nature documentary (realistic condition). The film presentations lasted about 20 min and were carried out simultaneously in two separate rooms. After the film presentations, the two groups were combined into one. The test session was carried out simultaneously with all the children in one large room. Each child sat at a separate desk. Because the experiment was carried out in several preschools, the groups were small, and the researcher could oversee that the children did not imitate the responses of others. Moreover, the children from the two comparison groups were tested together, so consulting responses would work against the hypothesis that the groups differ in their inferences.

The researcher presented children with the term “axon,” describing it as “a little tube in the form of a tree. Axons are narrow tubes that carry information.” After that, the experimenter presented a picture of a lizard and asked the children to name it.

The experimenter then attributed the target feature to a lizard by saying “A lizard has lots of axons inside. We can’t see them because they are inside the lizard. Let’s repeat the word, that we learned today, AXON. Great. Lizards have axons inside, but maybe not just the lizards?” Following this introduction, the experimenter distributed the answer sheets with photos as well as Post-it strips. Children were given the following instruction: “There is a sheet in front of you with lots of pictures. Look at the pictures and think if anything on them has axons just like the lizard. Use the sticky papers to mark the pictures of objects that you think have axons just like the lizard.”

**FIGURE 1:**
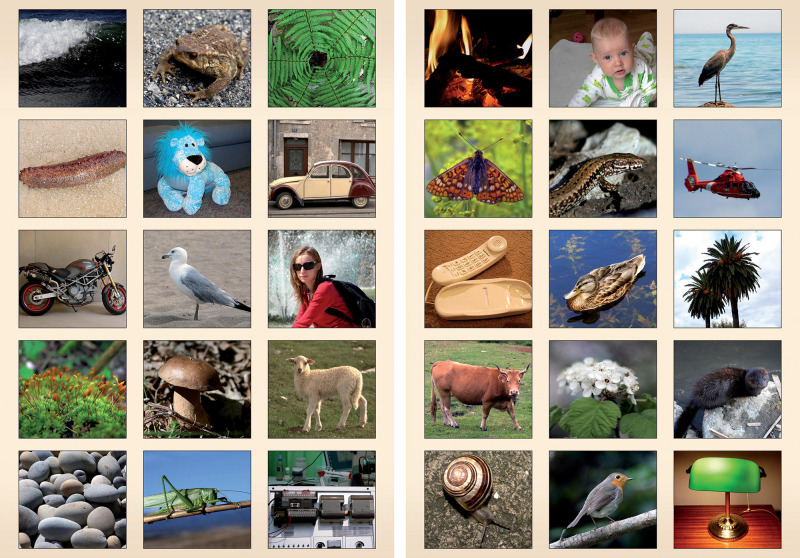
The display presenting 30 target items featured in the induction probe.

The children’s task was to attribute the target feature (axon) to items presented on the response sheet. The attribution of the feature to the item was coded as 1, while the omission of the item was coded 0. There were eight response categories including (1) humans, (2) mammals, (3) birds, (4) reptiles and amphibians, (5) invertebrates, (6) plants and fungi, (7) non-living natural kinds, and (8) artifacts (see Figure 1). Individual child’s responses to items belonging to each of the eight categories were averaged. The proportion scores were used in the analysis.

## Results

With the use of the SPSS software, we carried out an ANOVA with the proportion of attributions as a dependent measure, the item category as a within subject variable, and a comparison group (cartoon v nature documentary) as a between-subjects variable. The initial analysis included gender as a between-subjects variable but it did not show any significant effect or interaction of gender with the remaining variables, therefore gender was dropped from further analyses.

**FIGURE 2:**
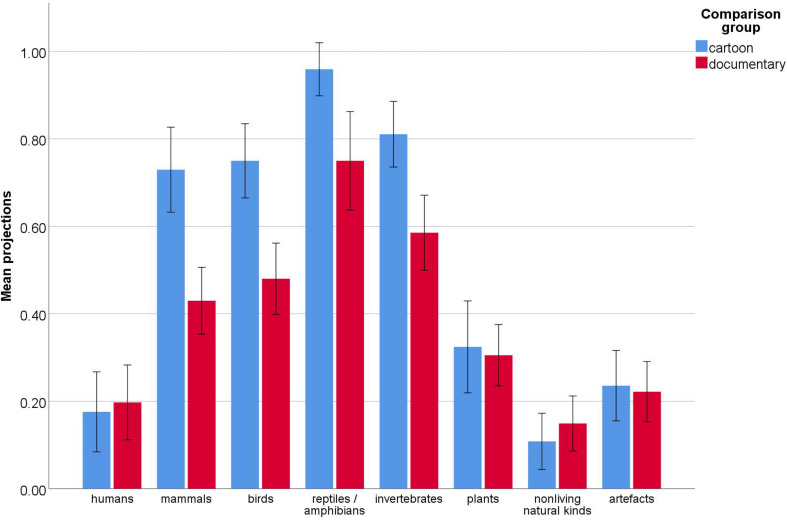
Proportions of attributions of axons by target category and comparison group. Error bars represent 95% confidence intervals adjusted for repeated measures.

The analysis yielded an effect with the comparison group *F*_(1, 73)_ = 5.34, *p* = 0.02, partial η^2^ = 0.07. The cartoon group made broader projections overall than the documentary group (*M* = 0.51, *SD* = 0.16 vs. *M* = 0.39, *SD* = 0.28). Due to non-sphericity, the Greenhouse-Geisser correction was applied to repeated measures results. There was an effect of category, *F*_(5.6,410)_ = 76.44, *p* < 0.001, partial η^2^ = 0.51. A series of within-subject least significant difference (LSD) *post-hoc* comparisons showed that children projected more to all non-human animals than to the remaining categories. They did not differentiate between those other categories, projecting equally to humans, plants, artifacts and non-living natural kinds. Within non-human animals, children made significantly more projections to reptiles/amphibians than to all other categories. They made more projections to invertebrates than to mammals, and they did not differentiate between birds and mammals. All tests were two-tailed with a significance level of 0.05. The analysis also yielded a significant interaction between category and comparison group *F*_(5.6,410)_ = 5.30, *p* < 0.001, partial η^2^ = 0.07. A series of between-group LSD *post-hoc* comparisons revealed that the cartoon group made significantly more attributions to the bird, mammal, invertebrate, reptile/amphibian category than the documentary group did, but there were no significant between-group differences for the remaining categories. All tests were two-tailed with a significance level of 0.05 (see Figure 2).

As part of the follow-up on the interaction effect, we carried out a series of within-subjects LSD *post-hoc* comparisons between animate categories excluding humans, separately for each comparison group. The documentary group projected more to reptiles/amphibians than to any other animate group and they also projected more to invertebrates than to mammals. The cartoon group also made more projections to reptiles/amphibians than to the remaining categories but no other difference was statistically significant. All tests were two-tailed with a significance level of 0.05.

## Discussion

In this study, we asked whether presenting children with the film depicting a variety of animal species which display either uniformly anthropomorphic or diverse, species-specific behaviors (cartoon vs. documentary), affects the extent to which children consider animals to be internally similar. As a measure of perceived internal similarity, children were asked to project an internal, invisible feature, axons, from a specific animal, lizard, across a broad set of items, including animals representing diverse taxonomic groups. We assumed that strong projections to all the test animals, irrespective of their taxonomic or perceptual similarity to the base, constitute an indication of children’s construal of animals as internally similar. Conversely, narrow, restricted projections would constitute a sign that children perceive animals as internally variable.

The findings that we obtained are very straightforward and suggest that witnessing various animals behave in a similar way leads to perceptions of greater internal homogeneity within non-human animals. The comparison groups differed in the strength of inferences within non-human animates. Children in the cartoon group made significantly more projections to all the categories of non-human animals than children in the documentary group. The two groups did not differ in how they differentiated between the categories of non-human animals. That is, both groups projected more strongly to reptiles/amphibians. Moreover, both groups showed the same undifferentiated rate of projections to all categories outside the non-human animal realm. It is worthy to note that both groups uniformly rejected humans as possessing the feature of the lizard. Their projections from a lizard to humans were no different than their projections to inanimate objects.

In order to fully understand the key finding in this study, it is necessary to address several issues: the influence of unrealistic content on knowledge about real animals, the mental process mediating the transfer from the documentary or the cartoon to the inductive task, the perceived place of humans within the animal category, and the relative influence of the two materials on induction.

First of all, the cartoon features a fictitious story with animals displaying impossible traits. The question arises, how such a content may influence children’s representation of real animals as tested in the subsequent induction task. Fictitious stories contain a mixture of realistic and unrealistic information. The task of distinguishing between these types of information is called a reader’s dilemma (Hopkins and Weisberg, 2017). Weisberg and Hopkins (2020) show that whether children are willing to extend information within a fictitious story and export it from the story to reality depends mostly on the kind of information. They seem to be more willing to export information that they consider probable, and they are well aware that anthropomorphic features of animals presented in the stories do not extend to the real world. Sutherland and Friedman (2013) also showed that children are selective about the information they extend from pretend play to real life. They are more likely to extend the information that is plausible than the information that is implausible. However, unlike Sutherland and Friedman (2013) or Weisberg and Hopkins (2020), who probed direct transfer of the specific features presented in the story, the present study probed a much more indirect effect of fictitious and realistic materials. Evidence that children’s real-life knowledge is indirectly affected by fictitious content comes from the study by Waxman et al. (2014). In their study, inductive inferences of realistic features to real animals differed between children who were presented with the anthropomorphic story and those who were presented information from an animal encyclopedia, despite the fact that the content of reading had no direct bearing on the inductive task.

Second, the extent of transfer from the films to the test task is an important issue that needs to be analyzed. Children watched films featuring various animals displaying anthropomorphic or realistic behaviors. There was no mention in the films of the animals’ internal structure. Yet, in the test task, children were asked to project internal features. Because the manipulated content and the test task are conceptually distinct, the explanation of the results requires an account of the reasoning process that made the transfer possible.

Children engage in various types of reasoning when they strive to understand the natural world. They solve problems, interpret data, explain cause-effect relationships, explain phenomena which require making a connection between existing and new knowledge during the (flexible) transfer of knowledge to meet the demands of novel situations (e.g., Mayer, 2002; Schönborn and Bögeholz, 2009). Transfer itself can be specific or unspecific (transfer of specific content knowledge or skills to new situations vs. transfer of strategies or principles to other contexts), positive or negative (whether it facilitates or inhibits learning) as well as proximal and distal (“small” vs. “large” transfer requirements). There can also be ‘horizontal’ and ‘vertical’ transfer (formulating generalizations within the same level of complexity or at a super-ordinate level) (Hasselhorn and Mähler, 2000). In the present study, the transfer from diverse or similar behaviors to heterogeneous or homogeneous internal structure can be considered positive, distal, horizontal, and unspecific. Such transfer can occur if there is an underlying explanatory principle organizing the domain.

One such explanatory principle organizing the domain of living things is psychological essentialism. According to psychological essentialism proposal, people expect living things to possess essences, which are intrinsic internal properties endowed with causal powers. Gelman (2003, p. 61) argues that people construe an essence as an invisible and internal property “having the capacity to influence outward behaviors and preferences.” Essentialism is believed to be an early emerging construal. There is ample evidence that young children hold essentialist expectations about living things (Gelman, 2003). If children expect that there is a causal link between internal composition and behavior such that the insides of an animal are responsible for its observable actions, then they may be willing to make inferences from homogeneity or diversity of behavioral patterns to internal composition.

Children link insides with behavior at a very early age. Many 8-month-olds assume that items displaying animacy cues and self-generated motion are not hollow (Setoh et al., 2013), whereas 14-month-olds expect that objects with similar insides also behave in similar ways (Newman et al., 2008). At an older age, the expectations about the links between behavior and internal structure become quite specific. Many 4–5-year-olds assume that the recipients of a heart transplant would display behaviors characteristic to the donor, both in case of within-species (humans) and between species (pig-monkey) transplant (Meyer et al., 2017). These data show that children expect specific kinds of behaviors to originate from essential, internal properties. There is also data showing that children make inferences from behavior to insides, as is the case in the present study. Gelman (2003) reports that 4-year-olds made general inferences from behavior to insides when they attributed a heart and muscles to animals without animacy cues (lacking eyes or faces) if they moved by themselves. The inference from behavior to insides in the domain of artifacts is evidenced by Ahl and Keil (2017), who show that 6–7-year-old children infer that machines displaying more diverse functions possess more complex insides.

In light of the studies reviewed above, it can be hypothesized that children who witness diverse sets of animals behaving in a similar way, by virtue of essentialist construal of living things, expect the congruent behavior to be driven by common internal composition. Consequently, it is natural to expect that the dimension of behavioral diversity/homogeneity within a category, translates into the expectation of internal diversity/homogeneity, which manifests itself in the pattern of inductive inferences.

It must be noted that there is a simpler explanation of the present results than the one invoking essentialism. The similarity of behavior in the cartoon and distinctive behavior in the documentary could be temporally encoded in similarity metrics performed between animals during the induction study. This would require the perceptual representations of animals presented as photographs in the induction study to be infused with the behavioral information from the films. The present study does not contain sufficient evidence to disentangle between these alternatives. It would be worth following up the study with tests of perceptions of similarity performed on photographs of objects that were previously observed as behaving in similar or distinctive ways.

A challenge to establishing a relationship between behavioral similarity and inductive inferences of internal properties comes from Heit and Rubinstein (1994). One of their findings is that inferences about anatomical properties are not informed by behavioral similarity. That is, knowledge of shared behavior between premise and conclusion categories did not increase the likelihood of internal property projections. In the present study, we make a seemingly contradictory claim that priming children with behavioral similarities strengthens their internal property projections. It is necessary to explain why Heit and Rubinstein’s (1994) findings may not contradict our interpretation of the present results. First of all, Heit and Rubinstein’s (1994) participants, as adults, distinguish behavioral similarities which result from either common descent or convergent evolution. Only in the first case, behavioral similarity stems from internal commonalities. Moreover, Heit and Rubinstein’s (1994) participants drew from their existing knowledge to make novel inferences. However, in our study, children were primed with behavioral similarity before making novel inferences. Heit and Rubinsten’s (1994) findings do not speak to whether participant’s judgments of internal commonalities could or could not be increased by learning of a novel behavioral commonality, particularly in participants who did not represent the distinction between convergent evolution and common descent. Finally, behavioral commonalities between pairs in Heit and Rubinstein (1994) were ecological adaptations such as diet or locomotion that were specific to a subset of animals. In contrast, behavioral commonalities primed in our study encompassed the entire animal kingdom.

One interesting issue that needs to be addressed is the very low rate of projections from the lizard to humans for both comparison groups. If the cartoon increases the expectation of greater internal similarity within animals, the cartoon group should also make more projections to humans. A plausible explanation of why this was not the case can be found in Carey’s (1985) analysis of childhood anthropocentrism. First of all, Carey notes that young children vehemently deny that people are animals. Second, children see humans as a preferred knowledge source about animals and they treat the instruction as a Gricean implicature, that is, a message in which explicit content differs from intended meaning (Davis, 2019). It is possible, that children excluded humans from their projections, because they may have assumed that the experimenter implied it was not a human feature by teaching it on lizards. By this reasoning, if humans possessed the target feature, it would be introduced as a human rather than lizard feature. If this explanation were true, this would point to the population tested as characterized by trait anthropocentrism.

An important outstanding issue is gauging the relative role of the two films in influencing the inductions. The inclusion of a control group (a condition in which children do the experimental induction task without previously being exposed to any material about animals) in the design could constitute a step in elucidating the mechanism of influence of the two types of film on induction. In the simplest way, by comparing the two experimental groups to the baseline, we would know whether the difference in responses is mostly driven by the influence of the cartoon or the documentary. A control group could also shed light on a possibility that a conspicuous grouping feature present in one of the films led to the observed difference in projections. However, the inclusion of the baseline could be misleading given the high variability of inductive inference patterns relative to children’s prior experiences with nature (Ross et al., 2003; Tarlowski, 2006, 2017).

The proximity of the baseline to either of the two experimental groups would be highly dependent on children’s prior experiences with animals. The children participating in this study did not represent an extreme of the poor/rich nature experience continuum. Despite being urban, they received an extended nature curriculum that included much experimentation and hands-on experience with living kinds. Any meaningful comparison to the baseline should be done by controlling for the diversity of experiences with nature. Based on the present results, it is clear that future research should address the question of how prior knowledge interacts with exposure to various types of fictitious and realistic depictions of animals in the formation of children’s representation of the living domain.

Finally, we would like to address the implications of the present study for biology education. The results of the present study might indicate that providing children with anthropomorphic representations of varied species is beneficial when the learning goal is to show children the unity of life, and explain that, in some ways, all living organisms share several features and activities, or simply to support students in making generalizations. Especially that, as it was shown in several research studies, anthropomorphism has the potential to aid conservation biologists conserve target species and stimulate wildlife value shift and change attitudes toward wildlife into more pro-environmental view (Chan, 2012; Manfredo et al., 2020). On the other hand, when the teaching goal is to emphasize the diversity, variety of life it may be recommended to rely on realistic materials presenting diverse species, such as the nature documentary shown to children in the present study.

## Data Availability Statement

The raw data supporting the conclusions of this article will be made available by the authors, without undue reservation, to any qualified researcher.

## Ethics Statement

Ethical review and approval was not required for the study on human participants in accordance with the local legislation and institutional requirements. Written informed consent to participate in this study was provided by the participants’ legal guardian/next of kin.

## Author Contributions

AT created the initial idea and the design of the study, contributed to the theoretical analysis as well as the introduction and discussion sections, analyzed the results, and wrote the results section. ER contributed to the theoretical analysis, the introduction and discussion sections, and carried out the final editing of the entire manuscript. Both authors contributed to the article and approved the submitted version.

## Conflict of Interest

The authors declare that the research was conducted in the absence of any commercial or financial relationships that could be construed as a potential conflict of interest.
